# Normalisation of AHI Under Positive Pressure Therapy Does Not Necessarily Mean Control of Symptoms: A Comparison of the Effectiveness of APAP and CPAP on Daytime Sleepiness and Nocturnal Urination

**DOI:** 10.3390/life15060969

**Published:** 2025-06-18

**Authors:** Sorin Bivolaru, Ancuța Constantin

**Affiliations:** 1 Faculty of Medicine and Pharmacy, University “Dunărea de Jos”, 800008 Galați, Romania; 2 Pneumology and Somnology Department, Military Emergency Hospital “Aristide Serfioti” Galati, 800150 Galati, Romania; 3 Department of Pneumology II, Division of Cardiothoracic Pathology IV, University of Medicine and Pharmacy “Carol Davila”, 050474 Bucharest, Romania; ancuta-alina.constantin@umfcd.ro; 4 Institute of Pneumology “Marius Nasta”, Medical Department III Pneumology, 050159 Bucharest, Romania

**Keywords:** obstructive sleep apnea (OSA), continuous positive airway pressure (CPAP), positive airway pressure (APAP), arousal index, sleepiness, wakefulness inability, fatigue, adherence

## Abstract

In practice, many patients receive APAP treatment for the simple reason that it provides increased comfort and is easier for patients to accept and tolerate. Reality has proven that we have very many patients diagnosed with OSAS on APAP treatment, under which AHI has normalized, but patients continue to have remaining symptoms. Thus the question was born: is the persistence of remnant symptomatology under APAP related to the mode of ventilation in patients with normalized AHI? The target group was young obese men presenting to the urology service for nocturnal pollakiuria without urologic cause. After performing nocturnal ventilatory polygraphy, the patients were recommended APAP treatment for three months, subsequently, the patients were switched to CPAP treatment for another three months, thus comparing the results obtained. After 6 months of treatment, 71.4% of the subjects would opt to continue CPAP treatment. While a clear option for APAP treatment was expressed by 10.2%. Our research, suggests that we should not be misled by the normalization of AHI under APAP therapy, but to evaluate the patients also with the help of available and standardized questionnaires.

## 1. Introduction

Obstructive Sleep Apnea Syndrome (OSAS) is the most common and well-known chronic sleep disorder in which complete or partial airway obstruction, caused by pharyngeal collapse during sleep that manifests as excessive daytime sleepiness, which is associated with increased cardiovascular and metabolic morbidity and mortality [1,2]. However, most patients are not aware of and do not present to their doctor for daytime sleepiness, but for nocturnal snoring and pauses in breathing during sleep observed by their bed partner or family. Sometimes they present to the doctor for other associated symptomatology such as frequent nocturnal urination, as in our analysis. Nocturnal snoring and sleep apnea being among the most characteristic and suggestive symptoms for OSAS. Other symptoms commonly seen in patients with OSAS are: morning headache, hypertension, memory disturbances, mental irritability, nocturnal pollakiuria, muscle spasms, excessive night sweats, erection disturbances, nocturnal physical restlessness, nightmares, etc. [3,4,5,6,7].

The natural and desirable treatment for OSAS in overweight or obese patients is weight loss, but until this is achieved, the standard recommended treatment is continuous positive airway pressure (CPAP) administered to the airways using a nasal or oronasal mask [8,9]. Positive pressure therapy acts like a pneumatic splint on the soft tissues of the upper airway, thus preventing their collapse and limiting or completely cutting off airflow during sleep, thus improving the quality of life of these patients by eliminating or significantly reducing snoring, completely eliminating pauses in breathing during the night, with important implications for reducing daytime sleepiness and nocturnal urination.

After the diagnosis of OSAS, in order to eliminate residual snoring and airflow limitations in the upper airways (apnea and hypopnea), patients are indicated for CPAP treatment [10]. However, in practice [11,12,13,14], many patients receive APAP treatment for the simple reason that it provides increased comfort and is therefore easier to accept and tolerate by patients [15,16]. The objective of the study was to compare the impact of CPAP versus APAP on daytime somnolence and nocturnal urination, as the subjects were entered into the study at the indication of the urologist, where patients presented for nocturnal reno-vesical symptoms [9].

## 2. Materials and Methods

### 2.1. Study Design

The present study is a comparative crossover study to compare the response of APAP versus CPAP to daytime sleepiness and nocturnal urination in young but obese patients.

### 2.2. The Motivation of the Present Analysis

Many of the patients diagnosed with OSA on APAP therapy, with adherence to treatment greater than 80%, i.e., they slept more than 80% of the monitored nights on APAP therapy, nights in which they had an average nocturnal sleep duration under therapy of at least 6 h, where the APAP device report indicated a normalized AHI. Although the AHI was normalized under APAP therapy (AHI < 5/h), patients recognized in the discussion card with the examining physician, the persistence of some OSA-specific symptoms—daytime sleepiness and nocturnal urination, but at a lower intensity compared to the period before starting APAP therapy. In this context, questions arose about the efficacy of APAP therapy on symptomatology, given that APAP positive pressure therapy normalized AHI. In order to be able to identify a link between the APAP ventilation mode and the remaining symptoms—daytime somnolence and nocturnal urination, we aimed primarily to compare the two types of therapy in young but obese patients recruited through the urology service, where they presented strictly for frequent nocturnal urination.

### 2.3. Patient Selection

This was conducted strictly through the urology service, where patients presented for nocturnal pollakiuria. Thus, 49 young, obese men with no known medical history were included in the analysis. The major selection criteria were: nocturnal pollakiuria without urologic cause, identified in young obese patients. The rest of the OSAS-specific symptomatology was identified anamnestically and with specific questionnaires. After a complete urologic evaluation—the usual tests, urinalysis, uroculture, rectal cough, PSA, Free PSA, prostate ultrasound before and after urination and uroflowmetry—and the confirmation of a urologic pathology underlying the frequent nocturnal urination, the urologist raised the suspicion of OSAS and the patients were referred to the somnology department for further investigations with nocturnal ventilatory polygraphy.

### 2.4. Exclusion Criteria

To increase the accuracy of the comparison between APAP and CPAP therapy, we excluded from our analysis elderly patients with comorbidities: cardiac, metabolic, respiratory, neurologic, oncologic, urologic—benign prostatic hypertrophy, etc. We took into account that these categories of patients may have organic or medicinal causes for nocturnal urination, such as: benign prostatic hypertrophy or diuretic treatments (furosemide, indapamide, spironolactone), which may frequently lead to nocturnal urination due to diuretic treatment in the evening or before bedtime, thus contributing to decreased accuracy of the study. In addition, according to the inclusion criteria, we selected only patients coming from the urology service, where they presented strictly for frequent nocturnal urination, the rest of the OSAS-specific symptoms being identified later during the discussion between the somnologist and the patient.

### 2.5. The Recruitment

The period of the 49 subjects included in the analysis was 2 years (2021–2023), and the follow-up period was 6 months from the diagnosis of OSAS—three months for APAP positive pressure treatment and three months of CPAP positive pressure treatment. Basically, after patients were recruited by the urology service according to the mentioned criteria, OSAS-specific symptomatology, identified during the punctual anamnesis, patients were evaluated using the Modified EPWORTH Sleepiness Questionnaire, which contains a standard number of 8 questions, for which a maximum score of 24 points can be obtained. To these, the additional question was added by our coworker regarding the frequency of nocturnal urination. A score higher than 10 points indicates an increased risk of daytime sleepiness and the additional question was used to collect information on the frequency of nocturnal urination.

### 2.6. Therapeutic Intervention

After nocturnal ventilatory polygraphy, patients were recommended APAP positive pressure therapy for three months, subsequently, patients were switched to CPAP positive pressure therapy for three months, thus comparing the results obtained based on the Modified EPWORTH Sleepiness Questionnaire (MESS appendix 1) that patients completed on each visit to the doctor. An AlisNightONE polygraph was used for the diagnosis of OSAS and a Phillips DreamStation non-invasive ventilation machine was used for titration and initial persification. The same type of nasal mask—Phillips Pico Nazal—was used in all patients. At the time of diagnosis, at the end of three months of APAP positive pressure therapy and subsequently at the end of three months of CPAP positive pressure therapy, patients were reassessed using the Modified EPWORTH Sleepiness Questionnaire (MESS-anexa 1). It is worth mentioning that all patients included in the present analysis, before performing nocturnal ventilatory polygraphy to establish the diagnosis of OSAS, were evaluated by ENT, including nasal fibroscopy to rule out a medical pathology in this sphere.

In order to achieve the set objective, after the completion of the somnological investigations and the establishment of the diagnosis of OSAS, for increased comfort and good adherence to positive pressure therapy, all patients were initially recommended to use APAP positive pressure therapy at home with air administration through the nasal mask at pressures between 8 and 16 cm·H_2_O. With this pressure range, patients went home for a period of three months. At the end of the three-month period of APAP therapy, patients were reassessed using the Modified Epworth Sleepiness Epworth Questionnaire (MESS), the adherence ratio and the effectiveness of AHI therapy were checked, and then patients were switched to CPAP therapy for three months on the same mask and the same machine, with only the ventilation pattern changed. The CPAP pressure was set at plus 2 cm·H_2_O compared to the average recorded for each individual patient during the period of APAP therapy.

Example of the pressure setting of CPAP positive pressure therapy: If, during the three months of APAP positive pressure therapy the average pressure was 10 cm·H_2_O, for the three months of CPAP positive pressure therapy the positive pressure was set to 12 cm·H_2_O (10 cm·H_2_O APAP average plus 2 cm·H_2_O). At the end of the three-month CPAP positive pressure treatment period, patients were again monitored using the Modified Epworth Sleepiness Epworth Questionnaire (MESS) to check the adherence ratio and the effectiveness of CPAP positive pressure therapy using AHI. Adherence to positive pressure therapy in this analysis required subjects to use APAP and CPAP for a minimum of 6 h per night, a minimum of 80% of the nights included in the 6-month follow-up period.

### 2.7. IBM SPSS Descriptive Statistics for Windows, Version 29.0. (30-Day Trial Version) Armonk, NY: IBM Corp.

Nominal data were presented as absolute frequency and percentage and continuous variables were expressed as mean, standard deviation, minimum and maximum. Independent samples *t*-test was used to compare the means by dichotomous variables in the study. ANOVA test was used to compare means of parameters between groups. A statistical significance coefficient *p* < 0.05 was considered significant.

## 3. Results

A total of 100 men were initially included in the analysis, according to the inclusion criteria, but only 49 of them were able to fulfill the criteria of adherence to treatment (80% of the nights slept under positive pressure therapy with an average of 6 h/night), rigorous and responsible accounting of the number of nightly urinations and attendance at the established follow-ups within the 6-month interval. Thus, a number of 49 young men aged between 32 and 55 years with a mean age of 44.92 years, with no significant medical history, who were selected strictly through the urology service, where they presented for reno-vesical symptomatology—nocturnal pollakiuria, were included in the final analysis.

All patients included in the present research were obese (mean BMI—39.62, minimum BMI—32.60, maximum BMI—52.33), which is why OSAS was suspected as the cause of nocturnal urination.

The route of administration of positive pressure therapy was as follows: first APAP, then CPAP. The rationale for this order was simple: firstly, to increase the adherence of the patients included in the study to positive pressure therapy, as it has been shown that patients tend to be more adherent to APAP positive pressure therapy. And secondly, APAP positive pressure ventilation helped us to identify the mean pressure below which the AHI normalized, thus representing the starting point for determining the positive pressure value of CPAP treatment.

By analyzing the evolution of AHI from the time of diagnosis (pre-positive pressure therapy) to the end of six months of treatment and follow-up, we find that the evolution is extremely good, with normalization of the apnea-hypopnea index (AHI) under both types of therapy, thus not identifying significant numerical and statistical differences between the two types of positive pressure therapy. Multiple studies collected sufficient data for comparative analysis of residual AHI during the use of APAP and CPAP positive pressure therapy. None of the studies reported a statistically significant difference in residual AHI between APAP and CPAP [8,17]. Analyzing Table 1 we observe that the mean AHI under the two types of positive pressure therapy—APAP and CPAP is within normal limits 5.33 (APAP) and 4.40 (CPAP) respectively, thus identifying no numerically and statistically significant differences between the two types of positive pressure.

Compared with the time of diagnosis, the AHI normalized under both types of positive pressure therapy and the MESS decreased significantly from 16.73 points pre-diagnosis (initial) of OSAS to 8.53 MESS points after three months of APAP positive pressure therapy, respectively to 3.22 MESS points after three months of CPAP positive pressure therapy (Table 2). Continuing the comparative analysis of mean sleepiness using the Modified Epworth Sleepiness Epworth Questionnaire (**MESS**) at the three time points of the study, the impact of each positive pressure therapy mode on sleepiness is shown. Of the two positive pressure therapy models, significantly better results were obtained under CPAP positive pressure therapy.

Analyzing the mean nocturnal urination, significantly better results were obtained by patients during the three-month period of CPAP positive pressure therapy, during which a mean of 0.92 urinations/night was calculated, compared to the baseline mean nocturnal urination of 5.55 urinations/night (pre-positive pressure therapy) and the mean value of 2.71 urinations/night calculated for the APAP positive pressure therapy period (Table 3). Analyzing the mean nightly urinations from the initial phase (pre-positive pressure therapy), also using the Modified Epworth Sleepiness Epworth Questionnaire (**MESS**), we observe that we have a mean value of 5.55 urinations/night, which decreases to 2.71 urinations/night after three months of APAP positive pressure therapy. Under CPAP, the results are significantly better. The mean nocturnal urination decreases to 0.92 at the end of the three-month treatment period, thus proving the superiority of CPAP positive pressure ventilation in this category of patients—young, obese, with frequent nocturnal urination.

After six months of follow-up during which all patients received positive pressure therapy according to the working method, 71.4% of the subjects included in the analysis said that they would definitely choose to continue CPAP positive pressure therapy. While, a clear option for APAP treatment was expressed by only 10.2%. Of the total of 49 subjects included in the analysis, nine subjects, or 18.4%, ticked both types of therapy, justifying their decision by the fact that they “felt equally comfortable with both therapies” (Table 4).

Analyzing separately the nine subjects (18.4%) using the Modified Epworth Sleepiness Questionnaire, who anamnestically felt no difference between the two types of positive pressure therapy, we find that eight (88. 88%) of the nine (100%) subjects significantly lower daytime sleepiness values were calculated under CPAP positive pressure therapy and in the case of the 9th (11.11%) subject, sleepiness calculated using the Modified Epworth Sleepiness Questionnaire was similar under both types of ventilation. Continuing the analysis of the nine subjects (9 = 100%) using the Modified Epworth Sleepiness Questionnaire, we find that the evolution of nocturnal urination under the two types of positive pressure therapies was significantly better during the period when patients used CPAP positive pressure therapy compared to the period when they used APAP positive pressure therapy.

Compliance data abstracted from ventilator card data identified similar treatment adherences for both types of positive pressure ventilation—APAP and CPAP. In both phases of the study, the same device was used to deliver both types of positive pressure. In order to avoid possible ‘patient attachment to the initial positive pressure’, patients were not told that the delivery of positive pressure would be completely changed, but were told that ‘a few’ adjustments would be made to the way the device would deliver the positive pressure so that the most appropriate type of positive pressure could be identified that would most effectively control sleepiness and night-time urination and therefore be most comfortable for the individual patient. As shown in Table 5 CPAP positive pressure therapy has a much better impact on sleepiness and nocturnal urination than APAP positive pressure therapy in this patient group. Analyzing the subjects’ choice at the end of the 6-month follow-up regarding the treatment modality, 71.4% of the subjects included in the analysis said that they would definitely choose to continue CPAP therapy. While a clear option for APAP treatment was expressed by only 10.2%. Of the total of 49 subjects included in the analysis, 9 subjects, or 18.4%, ticked both types of therapy, justifying their decision by the fact that they “felt equally comfortable with both therapies”. Analyzing the responses from the Modified Epworth Sleepiness Questionnaire and the ratings made by the nine subjects (18.4%) who opted for both types of positive pressure therapy, we find that eight (88.88%) of the 9 (100%) subjects had significantly lower daytime sleepiness values under CPAP therapy. Even though the sleepiness score decreased greatly under positive pressure therapy, it should be emphasized that a proportion of subjects still exhibit residual sleepiness, probably in the less elucidated area of the link between obesity and daytime sleepiness identified in non-apneic patients. Continuing the analysis of the nine (9 = 100%) subjects through the prism of the notes made during the follow-up period regarding the frequency of nocturnal urination, we find that the evolution was significantly better during the period when patients used CPAP positive pressure therapy, compared to the period when they used APAP positive pressure therapy, revealing once again the increased efficacy of CPAP positive pressure therapy on daytime and nocturnal urination in this category of men—young, obese, without known corneal diseases and who presented to the urology department for nocturnal urination—somnolence and nocturnal urination, even though AHI normalized under both types of positive pressure therapy.

## 4. Discussion

The most significant risk factor for developing obstructive sleep apnea (OSA) is weight gain and obesity. In addition, other recognized risk factors include: male sex, age over 40 in men and over 50 in women, and menopause in women. A neck circumference greater than 40 cm in men and over 37 cm in women is also associated with an increased risk. Other contributing factors are smoking, alcohol consumption, use of sedative drugs and a sedentary lifestyle. Importantly, although obesity is a major factor, it does not fully explain the higher prevalence of OSA in men. Localization of adipose tissue appears to play a more important role in the pathogenesis of the disease [18,19]. The prevalence of Obstructive Sleep Apnea Syndrome (OSA) varies considerably between studies, with estimates ranging from 2% to 10% of the adult population, and it is also increasingly common in children [15,20,21,22,23,24,25,26,27].

OSA is strongly associated with a wide range of comorbidities, including cardiovascular conditions such as ischemic heart disease, heart failure, hypertension, heart rhythm disorders and acute coronary syndromes. In addition, it is frequently associated with diabetes mellitus, gastro-oesophageal reflux disease, stroke and various endocrine disorders. This complex association emphasizes the urgent need for early diagnosis and treatment of OSA in order to prevent or effectively manage these concomitant medical conditions [1,2,28].

Studies have shown that APAP therapy increases adherence to treatment, but APAP ventilation algorithms may differ from one device to another, with the result that many APAP devices are not as effective as CPAP therapy [8,11,15,29].

Clinical experience has shown that a significant number of patients diagnosed with obstructive sleep apnea (OSA) and treated with automatic positive airway pressure (APAP) continue to experience daytime and night-time voiding (nocturia/polyuria), even after normalization of the apnea-hypopnea index (AHI). Articles published in the literature indicate that APAP therapy is as effective as continuous positive airway pressure (CPAP) therapy in normalizing AHI, improving sleepiness and improving patients’ quality of life [30,31].

However, the persistence of nocturia/polychuria in a considerable percentage of patients undergoing APAP therapy remains a relevant clinical observation. Given that the main etiopathogenesis of OSA is the collapse of the upper airway during sleep, the fundamental goal of treatment is to maintain its potency. This has direct implications for improving sleep quality, eliminating snoring, reducing daytime sleepiness and mitigating cardiovascular and neurological risks secondary to apnea, hypopnea and associated desaturations. This therapeutic goal is achieved by applying positive pressure, either by APAP or CPAP modality, through a nasal or oronasal mask [15,20,28,32,33,34,35].

The present work is the first study comparing APAP and CPAP positive pressure therapy in patients with very severe forms of OSAS, who were selected strictly through the urology service, where they presented for nocturnal urination. A complete urologic evaluation ruled out a renovesical disorder explaining the presenting symptoms, in which context the suspicion of OSAS was raised on the background of the mentioned symptoms and macroscopically evident obesity. Five women were also included in the study, but they did not show up for scheduled follow-ups or did not strictly adhere to the therapeutic and urinary monitoring recommendations and were therefore excluded from the analysis. The preponderance of information from already published research suggests that sleep-disordered breathing is more common in males. OSAS is more common in males due to the distribution of adipose tissue, upper airway anatomy, hormonal system, and polygraphic and polysomnographic monitoring. In this regard, it was much easier to select male patients for our analysis [25,36,37,38]. Regarding the elderly, their exclusion from the study was deliberate, given the associated comorbidities and the frame therapies that may frequently result in nocturnal urination (diuretic treatments) due to chaotic administration, thus increasing the uncertainty regarding CPAP and APAP therapies. The results of the present research showed that both types of therapy are equally effective if we restrict the evaluation of the results to the normalization of AHI alone, and the results are in agreement with multiple studies that have collected sufficient data for comparative analysis of residual AHI during the use of APAP and CPAP positive pressure therapy [31].

In previously published studies no statistically significant difference in residual AHI between APAP and CPAP therapy has been reported [8,17,33]. And the results of the present research showed that both types of therapy are equally effective if we limit the evaluation of the results only in terms of normalizing AHI. However, significantly better daytime sleepiness and nocturnal urination outcomes were accounted for at the end of the CPAP positive pressure treatment period. In order to be able to exclude patient attachment to the nocturnal breathing support device initially used [10,19], we decided to use only one type of device in the study, which can be programmed to operate in both APAP and CPAP mode. As mentioned above, at the end of the first three months of APAP positive pressure therapy, we decided not to disclose to patients that we would completely change the ventilation mode. Patients have been informed that “some” adjustments will be made to the way the device will deliver air over the next period.

According to the literature, the beneficial effects of positive pressure therapy (PAP) are predominantly limited to the period of nocturnal use of the device. It has also been shown that the benefits of this therapy can be quantified over a duration of 4-8 weeks. These findings supported the decision to establish a 12-week (3-month) follow-up period for each type of PAP therapy. This approach aimed to minimize overlap in quantification of the benefits of automated positive airway pressure (APAP) therapy during the first three months of the study with the continuous positive airway pressure (CPAP) therapy period, which spanned weeks 13–24 (i.e., months 4–6 of therapy and follow-up). We are aware that the possibility that residual benefits obtained during the CPAP therapy period (months 4–6) may also have been quantified during the APAP therapy period cannot be completely excluded. This potential influence may have contributed to improved outcomes in the CPAP group and we therefore recognize that this may be a limitation of the research. As mentioned previously, several published reviews have not identified major differences between APAP and CPAP therapy. However, in contrast to other reviews, the present research, in addition to using an innovative patient selection method, shows that in certain categories of patients, much better results are obtained under CPAP positive pressure therapy on daytime sleepiness and nocturnal urination. According to our research, patients diagnosed and treated with positive pressure (APAP or CPAP) for OSAS should not only be assessed by AHI normalizations, but also by standardized questionnaires regarding possible remaining daytime and nighttime symptoms under the initially chosen type of therapy. Depending on the outcome of the MESS, ESS or SWIFT assessment, somnologists should consider switching patients from APAP to CPAP positive pressure therapy [14,32,39].

In order to reach a consensus conclusion on the superiority of CPAP therapy over APAP therapy on nocturnal urination and daytime sleepiness, it is necessary to increase the sample size and the categories of patients studied.

Returning to the results of our analysis and analyzing the 9 subjects, who affirmatively “felt no difference between the two types of positive pressure therapy”, we find that 8 of the 9 subjects had significantly lower values of daytime sleepiness and nocturnal urination frequency during CPAP positive pressure therapy.

The results of the present study, in which the AHI normalized under both types of positive pressure therapy, reveal the importance of evaluating the efficacy of APAP or CPAP therapy also on the basis of standardized sleepiness questionnaires and not only by normalizing the AHI. Clearly, the small number of patients included in the present analysis requires its extension to a much larger number of patients, but also to other patient subgroups. Previously published trials have shown that APAP therapy increases adherence to treatment, however, APAP ventilation algorithms differ from device to device, leading to situations where many APAP devices are not as effective as CPAP therapy [29,40], which would require careful analysis and direct comparison of the effectiveness of different forms of APAP therapy [13]. To this, it is also worth adding that APAP ventilator pressure variations within the clinician-set pressure range in many patients induce microtreezures, fragmenting sleep sufficiently that patients continue to have residual daytime residual symptoms despite normalization of AHI [13,30,34,39,41].

In contrast to CPAP positive pressure therapy, APAP positive pressure therapy has the advantage of self-adjusting the pressure within the pressure range preset by the physician, depending on the modifiable parameters of OSA severity: weight fluctuations, alcohol consumption, continued smoking, body position during sleep or the use of medications and sedatives. However, in addition to the advantage of pressure self-regulation, APAP positive pressure therapy has multiple disadvantages, including: sleep disturbances and arousals induced by pressure variations. In addition to these two major disadvantages, the results of a study comparing the impact of APAP and CPAP on blood pressure control in patients with OSA, showed that APAP positive pressure therapy did not reduce 24-h diastolic blood pressure as effectively as CPAP positive pressure therapy [7,13,28]. Other studies have shown that APAP is not as effective as CPAP on sympathetic tone during sleep or on alleviating cardiovascular risk [13]. In the article [9,39,40], patients undergoing APAP positive pressure therapy were switched to CPAP therapy when it was found that they were not adherent, had symptoms remaining after the onset of APAP therapy, or complained of side effects of APAP therapy. After changing the ventilation mode and switching from APAP to CPAP therapy, patients showed improvements in adherence and sleepiness [39], and the results of our research are in agreement with the results obtained by the authors of the article [33,39], namely, CPAP therapy is superior to APAP therapy when it comes to daytime and nighttime symptom control [42]. Our research, as well as other research, suggests that we should not be misled by the normalization of AHI under APAP therapy, but assess patients for possible symptoms using available standardized questionnaires. Follow-up of patients after the end of the six-month period showed that all 49 subjects remained on CPAP positive pressure therapy even six months after the end of the follow-up period. In terms of possible adverse effects, subjects included in the study reported no incidents directly related to the therapy. The results obtained are similar to previously published evidence comparing the two types of positive pressure applied as treatment for patients with OSA [10,33].

However, there is a significant percentage of patients diagnosed with OSAS who refuse CPAP positive pressure therapy, and the reasons for this vary. In this context, the use of APAP-type positive pressure ventilation, where the pressure self-adjusts within the pressure range preset by the clinician according to the degree of resistance [17], may be considered, thereby improving patient comfort and compliance with therapy. The results of the present study open the opportunity to study OSAS patients from a subgroup perspective and call for further research on the scientific and practical status of the place of each of the two types of therapies in subgroups of OSAS patients. One of the major limitations of this study is the small number of patients and their selection on strictly urologic presentation criteria—frequent nocturnal urination and implicitly young men without associated comorbidities. All patients included in our analysis used APAP and or CPAP with the Philips Dreamstation ventilator, leaving open the question of the efficacy of APAP versus CPAP on other types of ventilators and raising the question: would the APAP positive pressure therapy algorithm used by other manufacturers be more effective under the conditions of the methodology chosen by us?

In addition to the exclusion of elderly patients and those with multiple comorbidities, and the absence of female subjects, another notable limitation of our study is the non-application of the multiple sleep latency test (MSLT) and the maintenance of wakefulness test (MWT). Also, another inherent limitation is the difficulty to exclude with certainty that the benefits of automatic positive pressure therapy (APAP) obtained in the first 12 weeks (3 months) of the study were not quantified in the subsequent period of continuous positive pressure therapy (CPAP), which could have led to apparently superior results under CPAP. This consideration is relevant in the context of published evidence indicating a quantification of the benefits of positive pressure therapies up to 4–8 weeks after treatment initiation, even though our study included 12-week follow-up periods. By ‘quantifiable benefit’ we mean improved control of the symptomatology targeted in the present study. The authors acknowledge that the standard study design would typically randomize the two PAP modalities used, which may have mitigated this limitation.

Smoking and alcohol consumption habits, together with the desire to lose weight, were not analyzed in the study as factors contributing to symptom control for at least two reasons: A: the number of those who quit smoking and alcohol consumption is very small and we considered that it would not be relevant for our analysis, which is why we, the authors, consider that further analysis in this direction is needed. And B: the patients analyzed in this study were all obese, and obesity is implicated in more than 80% of OSAS cases [25,43,44,45].

There is already a consensus that weight loss plays a major role in improving symptoms in OSAS patients. In addition, weight loss is a long-term process, and a possible correlation with symptom improvement would have been quantified only in the CPAP positive pressure CPAP treatment period, as this spanned the 4-6 month interval of our analysis. However, weight loss remains a very effective strategy for treating sleep apnea [25,26,27,43,44,45,46,47]. There are several studies in which investigators have demonstrated that a 10 to 15% reduction in body weight leads to an approximately 50% reduction in the severity of sleep apnea as assessed by (AHI) in moderately obese male patients [22,23,26,46,47].

**The particularity of the study is that in our analysis**—The ventilation pressure was set in the range of 8–16 cm·H_2_O for all patients, and the CPAP positive pressure was based on the average pressure recorded during the three months of APAP therapy, to which 2 cm·H_2_O was added. In most published studies, the APAP positive pressure was set “naturally” between 4 and 20 cm·H_2_O. A second peculiarity of the study was the method of patient selection, i.e., through the urology service where patients presented for frequent nocturnal urination, proven to be of non-urological cause.

## 5. Conclusions

In conclusion, current studies, including the present research, indicate that a proportion of patients with obstructive sleep apnea (OSA) with good adherence to positive pressure therapy (PAP) may have residual daytime and nocturnal symptoms, even in the context of a normalized apnea-hypopnea index (AHI). This observation emphasizes the need for a clinical approach that goes beyond simple AHI monitoring. Therefore, it is imperative that somnologists perform a detailed analysis of the individual patient’s symptomatology to determine which of the two modalities of therapy, automated positive airway pressure (APAP) or continuous positive airway pressure (CPAP), would be more effective in controlling residual symptoms. It is important to note that the American Academy of Sleep Medicine (AASM) has equated APAP and CPAP, recommending both as first-line therapies in the management of OSA. Although current evidence tends to position CPAP as the preferred therapy in some cases, particularly in patients with comorbidities, it is essential to recognize the significant role of APAP as a viable alternative, particularly for patients who have difficulty tolerating CPAP. The authors emphasize the need for further research to identify more specific and objective criteria to guide the choice of optimal therapy for various subgroups of patients with OSA.

## Figures and Tables

**Table 1:** Evolution of AHI are the two types of ventilation. Source: authors.

Date Validated	Initial AHI	AHI After Three Months of APAP Therapy	AHI After Three Months of CPAP Therapy
N	49	49	49
**Media**	**86.3**	**5.3**	**4.4**
Minim	56	1.2	1.2
Maxim	131	14.3	10.2
**Standard deviation**	1.9	3.3	2.2

**Table 2:** Comparison of mean sleepiness. Source: authors.

Date Validated	Epworth Originally Modified	Epworth Modified After Three Months of APAP Therapy	Epworth Modified After Three Months of CPAP Therapy
N	49	49	49
Media	16.7	8.5	3.2
Minim	11	2	0
Maxim	24	16	8
**Standard deviation**	3.4	3.6	2.1

**Table 3:** Comparison of mean nocturnal urination. Source: authors.

Date Validated	Average Night-Time Urination—Initial	Average Nocturnal Urination After Three Months of APAP Therapy	Average Nocturnal Urination After Three Months of CPAP Therapy
N	49	49	49
**Media**	**5.5**	**2.7**	**0.9**
Minim	3	0	0
Maxim	10	6	3
**Standard deviation**	1.8	1.5	0.9

**Table 4:** Representation of preference for positive pressure type. Source: authors.

APAP	Frequency	Percent
Yes	14	28.6%
No	35	71.4%
Total	49	100%
**CPAP**	**Frequency**	**Percent**
Yes	44	89.8%
No	5	10.2%
Total	49	100%
**APAP or CPAP**	**Frequency**	**Percent**
Yes	9	18.4%

**Table 5:** Comparison of sleepiness and night-time urination—APAP versus CPAP. Source: authors.

Daytime Sleepiness Under APAP	Daytime Sleepiness Under CPAP	Nocturnal Urination Under APAP	Nocturnal Urination Under CPAP
3	3	0	0
6	0	0	0
8	4	3	1
7	2	1	1
10	4	2	1
12	7	3	2
7	2	1	1
9	4	3	2
6	4	1	1
Media = 7.5	Media = 3.1	Media = 1.5	Media = 0.8

## Data Availability

The original contributions presented in this study are included in the article. Further inquiries can be directed to the corresponding author.
